# Assessment of HIV Post-Exposure Prophylaxis Use Among Health Workers of Governmental Health Institutions in Jimma Zone, Oromiya Region, Southwest Ethiopia

**DOI:** 10.4314/ejhs.v20i1.69429

**Published:** 2010-03

**Authors:** Bosena Tebeje, Chernet Hailu

**Affiliations:** 1 School of Nursing, Jimma University; 2 Department of Epidemiology, Jimma University

**Keywords:** post-exposure prophylaxis, health workers, HIV risk exposures, Jimma

## Abstract

**Background:**

Infection with Human Immunodeficiency Virus is a serious public health problem costing the lives of many people including health workers. Hence, Ethiopia has developed guideline on the prevention of infection in health institutions in July 2004 and also employed the use of post exposure prophylaxis since the implementation of free antiretroviral in January 2005. However in the country, specifically in Jimma zone, published studies showing the clear picture about HIV post exposure prophylaxis in the work place were non-existent. Therefore, this study was conducted to assess the knowledge, practice and factors associated to HIV post-exposure prophylaxis use among health workers of governmental health institutions in the Zone.

**Methods:**

A cross-sectional survey employing quantitative and qualitative methods was conducted from October to December 2008. Two hundred fifty four health workers participated in the quantitative study. Health workers for focus group discussion and key informants for in-depth interviews were identified with the help of administrators/ HIV/AIDs coordinators of the two administrative health bureaus and institutions included in the study. The quantitative data were entered and cleaned using Epi Info version 6.4 and analysed using SPSS for windows version 11.0. Descriptive statistics and chi-square test was employed to assess association among variables. P-value less than 0.05 was considered statistically significant.

**Results:**

Among the total 254 participants, 213 (83.9%) had inadequate knowledge about post exposure prophylaxis of HIV and 174 (68.5%) had ever been exposed to HIV risk conditions. Out of 174 health workers exposed to HIV risk, 105 (60.3%) sustained needle prick/cut by sharps, 77 (44.3%) to blood and 68 (39.1%) exposed to patients' body fluid. Perceived causes of exposure were; high workload 77 (44.3%), lack of protective barriers 58 (33.3%) and lack of knowledge on standard precautions 31 (17.8%). One hundred forty two (81.6%) of those exposed did not use post-exposure prophylaxis. Lack of information about the existence of post-exposure prophylaxis service 48 (33.8%), fear of stigma and discrimination 46 (32.4%), lack of understanding the value of reporting 33 (23.2%) and lack of support and encouragement to report 29 (20.4%) were the reasons for not using. Moreover, formal (separate) HIV post-exposure prophylaxis centre with proper guideline was non-existent in the study areas. Conclusions: In general, findings of the quantitative and qualitative study revealed that the knowledge of health workers about post exposure prophylaxis against HIV is inadequate. Though many of the studied health workers had HIV risk exposure, only few used post-exposure prophylaxis. Therefore, establishing a 24 hours accessible formal post-exposure prophylaxis centre with proper guideline is recommended. Health institutions are also advised to raise awareness of their employees on post exposure prophylaxis.

## Introduction

HIV/AIDs is a serious public health problem costing the lives of many people including health care workers (1). It is probably the most serious and causes the highest level of anxiety amongst health care workers (HCWs) in many countries including in Ethiopia. Ethiopia is one of the hardest hit countries by HIV/AIDS epidemic with the national HIV prevalence of 1.4% in adults (2). Each day thousands of healthcare workers (HCWs) around the world suffer accidental occupational exposures to blood borne pathogens (1). Preventing the occurrence of HIV infection resulting from such accidental injuries at work place and the use of HIV Post-exposure prophylaxis (PEP) is recommended by WHO/ILO (3). When administered shortly following exposure, PEP treatment has been shown to reduce the risk of HIV infection by 81% (4). Inline with this the Federal Ministry of Health of Ethiopia developed guidelines for infection prevention and PEP use (in the ART guideline) in 2004 and 2005, respectively (5, 6).

Providing relevant information on PEP for the health care professionals including managers would help to prevent the transmission of HIV, provide epidemiological data, identify unsafe practices, and reduce anxiety, and/or increase staff retention and productivity. However literatures evidenced that there is an information gap in the health care setups. For instance a study done in Guy's and St Thomas's hospital in London in 2001 indicated 93% of junior doctors had heard of PEP but fewer were aware that it reduced the rate of HIV transmission (7). A national study in Kenya also showed, among those who were knowledgeable, only 45% sought HIV PEP. The main reasons for not seeking PEP among this group was lack of sufficient information (35%) followed by fear of the process and what could follow (28%) (8).

In Ethiopia and specifically in Jimma zone, published studies showing the clear picture about HIV PEP in the work place were non-existent. Thus, this study was undertaken to assess the knowledge, practice and factors associated to HIV post-exposure prophylaxis use among health care workers of governmental health institutions in Jimma Zone and Jimma City.

## Methods and Subjects

Cross-sectional survey using quantitative and qualitative methods was conducted in Governmental Health Institutions in Jimma zone and Jimma City from October to December 2008. Governmental health institutions of Jimma area are under Jimma City and Jimma Zone Health Bureau. Health professionals who are directly involved in the care of patients in hospitals and health centres of the study area were the study population. Based on information from the bureaus, there were 569 health workers (218 (38.3%) in Jimma City and 351 (61.7 %) in Jimma Zone).

For the quantitative survey, a sample size of 265 was determined using Epi Info statistical soft ware version 6.4, Epitable calculator for single proportion using the assumptions: 5% desired precision, 50 % expected prevalence of HIV PEP use, 95% confidence level and 15 % non response rate due to the anticipated limitation of using self administered questionnaires.

Governmental health centres and hospitals in and out side Jimma City were considered geographically as two strata. The institutions in Jimma City included one hospital and two health centres while the institutions out side the city consisted of one hospital and 16 health centres. Then, representative sample of the health workers of different categories from the strata (112 from Jimma City and 142 outside the city), were included in the study using simple random sampling method since the number of the health workers was manageable for each discipline in each institution.

Purposive sampling technique used to select the key informants for the qualitative study. On the basis of the saturation level of the information, the study included 6 in-depth interviews of HIV/AIDS coordinators and 4 focus group discussions of health workers (2 each from the two strata) were conducted using topic guides and tape recorder. The transcribed and translated data of the In-depth/ focus group discussion (FGD) was analysed manually. The responses were tallied in the coding sheet, looked together and findings were summarised using computer by expanding responses to the fullest possible notes.

To ensure the validity and reliability of the data, the questionnaire and FGD guide were pre-tested in addition to giving training for research assistants (four nurses). The questionnaire consisted questions on socio-demographic, PEP of HIV knowledge and experience, and reasons for not using PEP.

Before data collection, ethical clearance and permission was obtained from Jimma University and respective health institutions authorities, respectively. Consent was obtained from participants and confidentiality of responses was ensured.

The research assistants distributed the selfadministered questionnaires, offered necessary instructions for the respondents to fill it anonymously and collected back questionnaires after checking for completeness and consistency of responses on each day of the data collection, under supervision of the principal investigators.

The quantitative data were entered and cleaned using Epi Info version 6.4 statistical package and analysed using SPSS for windows version 11. The qualitative data were tallied in the coding sheet, looked together and findings were summarised using computer by expanding responses to the fullest possible notes. In addition to descriptive statistics, chi-square test was employed to assess association among variables. P-value less than 0.05 was considered statistically significant.

**The following operational definitions and terms were used;**

*Adequate Knowledge- when respondents correctly answer ≥ 75 % of the eight knowledge questions.
*Inadequate knowledge- when the correct answer of respondents is < 75 % of the eight knowledge questions.
*PEP use /practice- reporting as they have practiced using Post-exposure prophylaxis of HIV.
*Post-exposure prophylaxis- is an emergency medical response that can be used to protect individuals exposed to the human immunodeficiency virus (HIV). PEP consists of counseling, laboratory tests and or medication (9).
*Exposure to HIV risk conditions-health workers' exposure to HIV risk conditions such as blood, patients /clients' body fluids, needle prick/sharps injury at work place.

## Results

A total of 254 health workers participated giving a response rate of 95.8%. One hundred eighty two (76.6%) were in the age group of 15–34 years, 136 (53.5%) females, 149 (58.7%) from health centres, 142 (56 %) working outside Jimma City and 94 (37.0%) had service year less than two years. Most, 173 (68.1%) of the participants were nurses and health assistants and 159 (62.6 %) earn a monthly income of 1000 and above Birr (Table 1).

**Table 1:** Socio demographic characteristics of respondent health workers of Governmental Health Institutions in Jimma Zone, Oct–Dec. 2008.

Socio demographic characteristics			Number (N=254)	percent
Age of respondents				
		15–24	92	36.2
		25–34	90	35.4
		35–44	47	18.5
		>44	25	9.8
Sex				
		Male	118	46.5
		Female	136	53.5
Place of work				
		Hospital	105	41.3
		Health Centre	149	58.7
Location of the Work place				
		Jimma City	112	44.1
		Outside Jimma City	142	55.9
Service year				
	Less than 2		94	37.0
	2–4		33	13.0
	5–7		46	18.1
	8–10		14	5.5
	11 and above		67	26.4
Field of profession				
Medical Doctor.			6	2.4
Laboratory Tech.			37	14.6
Nursing & health assist.			173	68.1
Health Officer			15	5.9
Midwife			23	9.1
Monthly income in Et. Birr				
500–999			95	37.4
1000 and above			159	62.6

The majority (83.9%) of the participants had inadequate knowledge about PEP of HIV risk exposure. On selected knowledge questions; measures to be taken after someone encounters needle prick injury at work place, measures to be taken when someone exposed to patients' blood, PEP reduces the likelihood of HIV infection after exposure and procedures of PEP of HIV exposure were answered correctly by 55.5%, 36.6%, 72.0% and 27.6% of the respondents, respectively (Table 2).

**Table 2:** PEP of HIV - knowledge of respondent health workers of Governmental Health Institutions in Jimma

Variables			Medical Dr. (n=6)	Laboratory Tech. (n=37)	Nursing / Health assist. (n=173)	Health Officer (n=15)	Midwife (n=23)	Total (n=254)
			N. (%)	N.(%)	N(%)	N(%)	N(%)	N(%)
**PEP of HIV Knowledge Level:**								
	Adequate knowledge		3(50.0)	5(13.5)	22(12.7)	3(20.0)	8(34.8)	41(16.1)
	Inadequate knowledge		3(50.0)	32(86.5)	151(87.3)	12(80.0)	15(65.2)	213(83.9)
**Response for selected knowledge** **questions:**								
PEP reduces the likelihood of HIV infection after exposure								
		Correct response	6(100.0) -	24(64.9)13	119(68.8)54	15(100.0)	19(82.6)4	183(72.0)71
		Not correct response		(35.1)	(31.2)	-	(17.4)	(28.0)
Measures to be taken after someone encounters needle stick injury at work place								
		Correct response	4(66.7) 2	20(54.1)17	96(55.5) 77	9(60.0) 6	12(52.2)11	141(55.5)
		Not correct response	(33.3)	(45.9)	(44.5)	(40.0)	(47.8)	113(44.5)
Procedures of PEP of HIV								
		Correct response	5(83.3)	9(24.3) 28	42(24.3) 131	5(33.3)	9(31.1)	70(27.6)
		Not correct response	1(16.7)	(75.7)	(75.7)	10(66.7)	14(60.9)	184(72.4)
Measures to be taken when someone exposed to patients' blood								
		Correct response	2(33.3) 4	14(37.8)	59(34.1)	8(53.3)	10(43.5)13	93(36.6)
		Not correct response	(66.7)	23(66.2)	114(65.9)	7(46.7)	(56.5)	161(63.4)

Zone, Oct–Dec. 2008.

Regarding exposure to the risk of acquiring HIV/ AIDs, 174 (68.5 %) of the 254 health workers reported to have been exposed to the HIV risk conditions. However, 142 (81.6%) of those exposed reported that they did not use PEP (Table 3).

**Table 3:** Health care workers exposure to HIV risk conditions and practice of PEP after exposure in Governmental Health Institutions, Jimma Zone, Oct–Dec. 2008.

Variables		Medical Dr	Laboratory Tech.	Nursing / Health Assistant	Health Officer	Midwife	TOTAL
No.(%)	No.(%)	No.(%)	No.(%)	No.(%)	No.(%)
Ever been exposed to HIV risk conditions (n=254)							
Yes		-	23(62.2)	123(71.1)	10(66.7)	18(78.3)	174 (68.5)
No		6(100.0)	14(37.8)	50(28.9)	5(33.3)	5(21.7)	80 (31.5)
Practice of PEP after exposure (n=174)							
	Yes	-	4(17.4)	26(21.1)	1(10.0)	1(5.6)	32(18.4)
	No	-	19(82.6)	97(78.9)	9(90.0)	17(94.4)	142(81.6)

Among the 174 health professionals exposed to the HIV risk conditions, the majority (60.3%) sustained needle prick or cut by sharps, 44.3% exposed to blood and 39.1% to patients' body fluid. The proportions of exposure to patients' body fluid among the different professionals differ significantly (P=0.002) (Table 4).

**Table 4:** Respondent health workers exposure to HIV risk conditions in Governmental Health Institutions, Jimma Zone, Oct–Dec. 2008.

Profession	HIV/AIDS risk conditions					
	Needle stick / exposure to sharps		Exposure to Blood		Exposure to body fluid	
	Yes No.(%)	No No.(%)	Yes No.(%)	No No.(%)	Yes No.(%)	No No. (%)
Laboratory Tech. (n=23)	16(69.6)	7(30.4)	6(26.1)	17(73.9)	1(4.3)	22(95.7)
Nursing & Health assist. (n=123)	71(57.7)	52(42.3)	57(46.3)	66(53.7)	52(42.3)	71(57.7)
Health Officer (n=10)	5(50.0)	5(50.0)	7(70.0)	3(30.0)	5(50.0)	5(50.0)
Midwife (n=18)	13(72.2)	5(27.8)	7(38.9)	11(61.1)	10(55.6)	8(44.4)
Total (n=174)	105(60.3)	69(36.7)	77(44.3)	97(55.7)	68(39.1)	106(60.9)

Needle stick /exposure to sharps, P.value = 0.444

Exposure to blood, P.value = 0.103

Exposure to body fluid, P.value = 0.002

The main reasons reported as a cause of exposure to HIV risk conditions in the work place were; high workload 77 (44.3%), lack of protective barriers 58 (33.3%) and lack of knowledge on standard precautions 31 (17.8%). The major reasons reported for not using PEP of HIV after exposure were lack of awareness of the existence of PEP service/ protocols by 48 (33.8%), fear of stigma and discrimination by 46 (32.4%), lack of understanding the value of reporting exposures by 33 (23.2%) and lack of support and encouragement to report by 29 (20.4%) (Table 5).

**Table 5:** Respondent health workers' perceived cause of exposure to HIV risk conditions and reasons for not using PEP of HIV in Governmental Health Institutions, Jimma Zone, Oct–Dec. 2008.

Variables	Health workers	
	Frequency	Percent
**Perceived cause of exposure to HIV risks :( n=174)**		
Lack of protective barriers	58	33.3
Lack of Knowledge on standard precautions	31	17.8
Heavy work load	77	44.3
Others	8	4.6
**Reasons for not using PEP: (n=142)**		
Unaware of the existence of PEP service and protocol	48	33.8
Lack of understanding the value of reporting exposures	33	23.2
Fear of stigma and discrimination	46	32.4
Fear of judgement from colleagues	6	4.2
Uncertain about confidentiality	15	10.6
Lack of support and encouragement to report.	29	20.4
The PEP service is far	16	11.3
Negligence	1	0.7
Client tested negative	5	3.5

Post exposure prophylaxis use was not associated with any of the workers' socio-demographic and other variables (P>0.05) (Table 6).

**Table 6:** Association of socio-demographic and other factors with HIV PEP use of health workers in Governmental Health Institutions, Jimma Zone, Oct–Dec. 2008.

Variables		PEP practice		X^2^	P-value
		YES	NO		
Age of respondents				
	15–24	11	52	3.497	0.321
	25–34	10	48		
	35–44	10	27		
	>44	1	15		
Sex					
	Male	15	65	0.013	0.910
	Female	17	77		
Place of work (Health institution)					
	Hospital	11	50	0.008	0.929
	Health Centre	21	92		
Location of the place of Work					
	Jimma City	12	51	0.028	0.866
	Jimma Zone (Outside Jimma City)	20	91		
Service year					
	Less than 2	6	50	4.794	0.309
	2–4	7	17		
	5–7	6	30		
	8–10	2	6		
	11 and more	11	39		

All discussants of the FGDs reported that PEP should be seen as a primary issue; otherwise the motivation and confidence of health workers could decrease. To the question about having information about PEP and source of information, some discussants responded that they had no information, some heard from friends informally and some other on training. One discussant said, “*we don't know where to go and why should we report*.”

The discussants also mentioned that lack of awareness of the existence of PEP, confidentiality problem, fear of stigma and discrimination, availability of provider initiated HIV counseling and test, that helps to determine the status of source patients and fear of ARV drug side effects as the causes for not to report/ resort for PEP.

Needle prick injury and blood splash for the majority and amniotic fluid for some were among the incidents encountered. Few of them replied that they faced these exposures while working in emergency units and being very busy. Some also responded “*we are caring for patients but no body cares for us. We lost many of colleagues because of failing to use the PEP services*.”

Most of the discussants responded that risk exposure causes emotional stress, insecurity feeling (thinking what will happen to their family if they fall sick), reduce motivation and commitment to work. One participant expressed “*I hate my profession some times*”, the other one said “*I wish I were a driver or secretary…etc*”. Others replied, “*Heavy track drivers are considered as high risk but we are more at risk*”. The other said “*we devoted our life to our profession/patients but for us no body”. “I gave my life to God.”*

Six in-depth interviews involving HIV/AIDs focal persons of the two zonal offices, 2 hospitals and 2 health centers were carried out. All the coordinators/focal persons reported that there is no formal PEP center but they have designed procedures to entertain it. In Jimma University Specialized Hospital, for instance, effort was made to offer the service through matron office in 2007. Then, information letter about the availability of HIV/AIDs PEP was posted on in 2008. Since then when incidents encountered, source patients are tested in the same ward, then victims are sent to a ward offering ART prophylaxis based on the result. However there was no documented report on the number of incidents and PEP services given.

In other institutions attempt of referring incidents to ART and counseling centers was mentioned. In the district hospital outside Jimma town, the key informant mentioned that a committee was established to work on PEP issues. The reasons given for unavailability of PEP service in most of the health institutions of the study area outside Jimma town were lack of trained person, guideline and ART site expansion.

Almost all of the informants at different levels underlined the need to give ongoing training on HIV PEP, availing standard guidelines, referral linkages, and giving attention for the safety of health care professionals by the concerned authorities. Awareness creation, allocate separate fund, availing separate PEP center for confidentiality and convenience, proper documentation and reporting system and availing life insurance were also emphasized by the informants.

## Discussion

This study assessed the knowledge, practice and factors associated with HIV PEP use among health workers who are directly involved in the care of patients in the governmental health institutions of Jimma Zone and Jimma City, Southwestern Ethiopia.

Considerably low proportions of each category of the health workers were knowledgeable about PEP of HIV in this study area. The findings of this study are lower than the results of the study done in Malaysia Hospital where 56% of doctors and 25% of nurses were aware of correct risk of transmission of HIV at work place. And only few (1/10 of doctors and 1/3 of nurses) knew whom to contact immediately after injury (10). Similarly the study done in 2001 in Guy's and St Thomas's Hospitals, London indicated that only 8% of the doctors could name the drugs recommended in recent national guidelines and a significant proportion (43%) could not name any (7). Other literatures also supported that the knowledge about post-exposure prophylaxis among healthcare workers is poor (11, 12). This is an indicative of much work remained to be done to raise the awareness of health workers regarding PEP of HIV. The Indepth/FGDs of this study also substantiated the above issues.

Large number of health workers reported as they have ever been exposed to HIV risk conditions which is higher than the 2003 Italian study that indicated the overall (HIV, HCV, HBV) occupational exposure to be 11.3, 11, 4.9, and 4.1%, in midwives, nurses, cleaners, and laboratory technicians, respectively (13). This difference might be due to the difference in the settings.

Nevertheless, in this study, medical doctors reported that they had never been exposed to the risk of HIV. In contrary to this study, previous studies showed that considerable numbers of physicians were exposed to the risk of HIV. The study in Guy's and St Thomas's hospitals, revealed 76% of junior doctors had experienced high risk of exposure to potentially infective material at some stage in their careers but only 18% had sought advice about PEP following potential exposures (7). The 2003 Italian study also indicated that the overall (HIV, HCV, HBV) occupational exposure to be 12 and 3.9%, in Surgeons and physicians, respectively (13). Similarly, the study of almost 700 surgeons-in-training at 17 US medical centers found that 582 (83.1%) had experienced a needle stick injury (14). This difference might be due to the presence of social desirability bias in the present study or doctors might have used universal precautions better than others. The later explanation also can be applied for the exposure of lesser proportion of the health workers to needle prick/cut by sharps in the current study than the finding documented in the study done in Nepal in 2003 (15).

The quantitative and qualitative study revealed similar results on perceived causes of exposure of health workers to HIV risk conditions in their work place and were also supported by the result of the study done in Johannesburg University (16).

Like the Nepal study finding most exposed health workers didn't use PEP (14). Similarly, 297 of the 578 most recent incidents had not been reported to an employee health service, including 15 of the 91 cases involving high-risk patients in the US study (14).

In this study, the major perceived reasons reported for not using PEP of HIV after exposure were almost similar with the findings of the studies done in Australia, Kenya and others that identified the reasons which discourage reporting of the risk of an HIV occupational exposure including fear of reprimand, uncertainty regarding the confidentiality of the results, being unaware that a protocol exists for reporting and dealing with occupational exposure, and lack of support and encouragement to report (1,8,17,18).

In conclusion both the quantitative and qualitative study revealed that the knowledge and practice/use of health workers about post-exposure prophylaxis against HIV was inadequate. Majority of health workers do have exposure to the risk of HIV predominantly to needle prick and considerable proportion of health workers exposed to blood and body fluid. However, only few of them used PEP. Also formal HIV PEP centre with proper guideline/procedure were non-existent in the study area.

Therefore, the need to establish separate, 24 hours accessible, formal post-exposure prophylaxis centres, and proper guideline along with raising awareness were underlined. Moreover availing adequate resources /protective materials, adhering to standard precautions, and availing health life insurance for health workers at all levels including districts (Woreda) were recommended. Due to the obvious limitation of this study (cross-sectional study), doing further study, which is stronger in determining cause and effect relationship of the variables, is also advisable.
